# *Juglans regia* L. extract promotes osteogenesis of human bone marrow mesenchymal stem cells through BMP2/Smad/Runx2 and Wnt/β-catenin pathways

**DOI:** 10.1186/s13018-022-02949-1

**Published:** 2022-02-14

**Authors:** Xianlun Pang, Zhendong Zhong, Feng Jiang, Jian Yang, Hai Nie

**Affiliations:** 1 Health Management Center, The Affiliated Hospital (TCM) of Southwest Medicial University, No. 182, Chunhui Road, Longmatan District, Luzhou, 646000 Sichuan China; 2 Sichuan Provincial People’s Hospital, Chengdu, 610000 Sichuan China; 3 Department of Orthopedics, The Affiliated Hospital (TCM) of Southwest Medicial University, Luzhou, 646000 Sichuan China; 4 Department of Orthopedics, Yongchuan Hospital Affiliated to Chongqing Medicial University, Chongqing, 402160 China; 5 Department of Orthopedic Surgery, Sichuan Academy of Medical Sciences and Sichuan Provincial People’s Hospital East Campus, Chengdu, 610101 Sichuan China

**Keywords:** *Juglans regia* L., Osteogenic differentiation, Autophagy, BMP2/Smad/Runx2, Wnt/β-catenin, hBMSCs

## Abstract

**Background:**

The present study investigates the effects of *Juglans regia* L. (walnut, JRL) leaves extract on osteogenesis of human bone marrow mesenchymal stem cells (hBMSCs).

**Methods:**

hBMSCs were incubated with different concentrations of JRL extract (10, 20, 40, or 80 μM). Cell proliferation was evaluated by Cell Counting Kit-8 assay (CCK-8) assay. ALP activity and Alizarin Red staining were used to assess the osteogenesis of BMSCs. Western blot was performed to measure the levels of proteins.

**Results:**

Our results showed all concentrations of JRL extract had no significant effect on cell proliferation. JRL extract concentration-dependently promoted osteoblastic differentiation and cell autophagy of hBMSCs, characterized by the increased expression of pro-osteogenic markers alkaline phosphatase (ALP), osteocalcin (BGLAP), osterin, and osteoprotegerin (OPG) and autophagy marker proteins (LC3II, Beclin-1, and p62). Furthermore, JRL extract stimulated the activation BMP2/Smad/Runx2 and Wnt/β-catenin signaling pathways in hBMSCs, which play key roles in osteogenesis differentiation. Meanwhile, BMP inhibitor (Noggin) and Wnt antagonist Dickkopf-1 (DKK1) both reversed the increases of BGLAP, osterin, and OPG expression induced by JRL extract.

**Conclusions:**

Our findings indicate that JRL extract regulated osteogenic differentiation and cell autophagy of hBMSCs through the BMP2/Smad/Runx2 and Wnt/β-catenin pathways.

## Background

Osteoporosis (OP) is a common senile disease, and its pathological features are characterized by decreased bone mass and destruction of bone micro-structure, resulting in decreased bone strength, increased fragility and increased risk of fracture [1–3]. Patients with spinal or hip fractures caused by OP may experience serious complications such as pulmonary infection and thrombotic diseases due to prolonged bed rest, which ultimately may result in fatal outcome [4, 5]. Currently, OP has become one of the senile diseases worldwide. Epidemiological investigations show that the risk of fracture in OP patients is as high as 40%, and about half of white women have OP after menopause [6]. As a serious complication of OP, the proportion of social public medical services expenditure caused by fractures has also gradually expanded. According to reports, 85% of the cost of fracture treatment is closely related to OP, and the cost of treatment it generates is more than 100 billion yuan nationwide [7]. Therefore, new targets for the early treatment of OP and the development of new drugs need to be further explored.

The dynamic balance between osteoblast-mediated bone formation and osteoclast-mediated bone resorption is the basis for maintaining the stability of human bone metabolism and the integrity of the body's bones. When the balance is disturbed, bone loss, bone density, and bone microstructure are destroyed, leading to increased bone fragility, and ultimately osteoporosis [8]. Of note, this imbalance arises from the imbalance between osteogenic and adipogenic differentiation of bone marrow mesenchymal stem cells (BMSCs) [9]. BMSCs can play a multidirectional and self-renewing function. They are widely distributed in bone marrow tissues of human. They can differentiate into osteoblasts, chondrocytes, adipocytes, myoblasts, and neurons under certain conditions [10]. There is a reverse change in the differentiation of BMSCs into osteoblasts and adipocytes, which is very important for the study of OP pathogenesis.

The treatment of OP is mainly based on drugs. The commonly used bone resorption inhibitors in clinical practice include bisphosphonates, selective estrogen receptor modulators, calcitonin, and estrogen [10]. Although various types of anti-osteoporosis drugs have achieved certain effects in clinical treatment, there are certain side effects. Bisphosphonates are commonly used in OP treatment, and bisphosphonate-associated osteonecrosis of the jaw (BRONJ) was a serious side effect [11]. Another type of OP drug, salmon calcitonin, was reported to have most common side effects of nausea and facial flushing [12]. Natural products have long been used to prevent and treat OP because they have fewer side effects. Previous studies have examined the efficacy of many plant active ingredients in the treatment of OP, some of which are resveratrol [13], emodin [14], quercetin [15], and milk thistle extract [16]. *Juglans regia* L. (walnut, JRL) is an annual herbal that belongs to the Juglandaceae family [17]. Its fruit can be eaten, and roots, stems, and leaves are used in folk medicine because of its large number of active ingredients [18]. The extracts from JRL were reported to inhibit oxidative injury [19], inflammation [20], and cancer cell proliferation [21]. Notably, previous in vitro research showed JRL extract-induced nodule formation in KS483 osteoblasts[22], implying that JRL could increase the activity of osteoblasts and had a beneficial effect on bone loss [22]. However, the effects and mechanisms of JRL extracts on osteogenesis of BMSCs have not yet been investigated.

In the present study, we aimed to determine the promoting effects of JRL leaves extract on osteogenesis and cell autophagy of hBMSCs by regulating BMP2/Smad/Runx2 and Wnt/β-catenin pathway, which play a role in osteoblast differentiation [23].

## Methods

### Extraction of plant material

The leaves of *Juglans regia* L. were collected from the city of Luzhou in Sichuan province (China). The leaves were shade dried and grind into powder. Then, the material was extracted with 500 mL of ethanol for 2 h using the Soxhlet apparatus. The supernatant was filtered and vacuum evaporated under pressure at 50 °C until a solid extract was obtained (18.2% yield). The resulting solid extract was stored at 4 °C for future use.

### Ultra-performance liquid chromatography–mass spectrometry (UPLC-MS) analysis

The chemical composition of JRL was analyzed by UPLC-MS. The UPLC-MS analysis was carried out with a hybrid Quadrupole-TOF LC/MS/MS Mass Spectrometer (B Sciex Instruments, Shimadzu LC30) using a Waters BEH C18 column (2.5 µm, 150 × 2.1 mm) with the column temperature at 40 °C. The mobile phase was 0.1% HCOOH-H2O (A)-acetonitrile (B) at a flow rate of 0.3 mL/min in gradient elution as follows: 0–2 min, 95% A; 2–13 min, 5% A; 13–15 min, 95% A. Electrospray ionization (ESI) positive and negative ion modes were used for detection. The ESI source conditions were set as follows: Ion Source Gas1 (Gas 1): 50, Ion Source Gas2 (Gas 2): 50, Curtain Gas (CUR): 25, Source Temperature: 500 ℃/450 ℃ (positive ion/negative ion), Ion Sapary Voltage Floating (ISVF) 5500 V/4400 V(positive ion/negative ion), TOF MS scan range: 100–1200 Da, product ion scan range: 50–1000 Da, TOF MS scan accumulation time 0.2 s, product ion scan accumulation time 0.01 s. The secondary mass spectrum was obtained by information-dependent acquisition (IDA) with high sensitivity mode.

### Cell culture and treatment

Human bone marrow mesenchymal stem cells (hBMSCs) were purchased from ScienCell (California, USA). The cells were cultured in DMEM medium supplemented with 10% fetal bovine serum (FBS; Gibco-BRL Life Technologies, Paisley, UK) and 1% penicillin/streptomycin sulfate (Shzeye, Shanghai, China), which is called as complete medium (CM), and then incubated in an incubator at 37 °C with 5% CO_2_ in a humidified incubator.

Cells were treated with one of different concentrations of *Juglans regia* L. (10, 20, 40, or 80 μM) extract in the presence of OIM (osteogenic induction medium, including DMEM, 10% FBS, 10 mmol/L β-glycerophosphate, 10 nmol/L dexamethasone, and 10 μg/mL ascorbic acid-2 phosphate) to identify the optimum concentration of JRL extract that induced the osteogenic differentiation of hBMSCs. Subsequently, cells were separated into 4 groups: PBS and OIM cultured group; JRL extract and OIM cultured group; OIM, JRL extract and 100 ng/mL BMP-inhibitory protein Noggin (MedChemExpress, HY-P7086) cultured group; OIM, LRL extract and 2 mg/mL Wnt antagonist Dickkopf-1 (DKK1, MedChemExpress, HY-P73513) cultured group. The grouping of experiments is shown in Table 1.

**Table 1:** The grouping of experiments (Exp)

	Group	Treatment
Exp. I	Control group	Osteogenic induction medium
	10 μM group	Osteogenic induction medium + 10 μM JRL extract
	20 μM group	Osteogenic induction medium + 20 μM JRL extract
	40 μM group	Osteogenic induction medium + 40 μM JRL extract
	80 μM group	Osteogenic induction medium + 80 μM JRL extract
Exp. II	Blank group	DMEM medium
	OIM group	Osteogenic induction medium + PBS
	OIM + 20 μM group	Osteogenic induction medium + 20 μM JRL extract
	OIM + 40 μM group	Osteogenic induction medium + 40 μM JRL extract
Exp. III	Blank group	DMEM medium
	OIM group	Osteogenic induction medium + PBS
	OIM + 20 μM group	Osteogenic induction medium + 20 μM JRL extract
	OIM + 20 μM group + Noggin	Osteogenic induction medium + 20 μM JRL extract + 100 ng/mL Noggin
	OIM + 20 μM group + DKK1	Osteogenic induction medium + 20 μM JRL extract + 2 mg/mL DKK1

### Cell survival assay

Cell viability was assessed using the Cell Counting Kit-8 assay (CCK-8; Boster, Wuhan, China) according to the manufacturer’s protocol. To be brief, cells were digested with trypsin (7 × 10^4^/mL) and seeded in 96-well plate (1.0 × 10^3^ cells/well). Then, the seeded cells were incubated at 37 °C and 5% CO_2_ for 48 h. Finally, the supernatant was removed. 100 μL CCK-8 solutions were added to each well and further incubated for 1 h at 37 °C. The absorbance values were read at a wavelength of 450 nm.

### Western blot analysis

Proteins extracted from hBMSCs cells were separated by 10% SDS-PAGE and then transferred to nitrocellulose membranes (Hybond, USA). The membranes were blocked with 5% nonfat milk and incubated with corresponding protein antibodies or a rabbit anti-β-actin monoclonal antibody. Then, the membranes were subsequently incubated with a HRP Goat anti-Rabbit IgG (Abcam, Cambridge, UK; cat. no. ab6721). The net optical density was analyzed with the gel Image processing system (Image-pro Plus 6.0). Primary antibodies were purchase from Abcam (Cambridge, UK) under the following item No: Osteocalcin (BGLAP; ab133612; 1:2000), Osterix (ab209484; 1:1000), OPG (ab73400; 1:1000), BMP2 (ab214821; 1:1000), Runx2 (ab76956; 1:1000), Smad1 (ab126761; 1:2000), p-Smad1 (ab226821; 1:1000), Wnt3a (Abcam, ab219412; 1:1000), β-catenin (ab32572; 1:5000), p-β-catenin (ab81305; 1:10,000), TCF-7 (ab134127; 1:1000), Lef1 (ab137872; 1:1000), DLX5 (ab32072; 1:1000), LC3II/LC3I, (ab192890; 1:2000), Beclin-1, and 962 (ab207305; 1:1000), and β-actin (ab8227; 1:1000). The bands were visualized using the chemiluminescence (ECL) system (Affinity Biosciences, Cincinnati, Ohio, USA).

### Detection of alkaline phosphatase (ALP) Activity

hBMSCs were cultured in 48-well plates under the optimal conditions of 37 °C and 5% CO_2_. After reaching 80% confluence, hBMSCs were treated with OIM or OIM containing different concentration of JRL (20, 40, or 80 μM) extract for 7 days. ALP activity was determined by the ALP colorimetric assay kit (Abcam, Cambridge, UK).

### Statistical analysis

The data were represented as means ± standard deviation (SD). The SPSS software (version 19.0, SPSS Inc., Chicago, IL, USA) was used to calculate all the values. One-way analysis of variance (ANOVA) followed by Tukey's-B post hoc tests were used for comparison between groups. The statistical significance was *P* < 0.05.

## Results

### Chemical composition of *Juglans regia* L. extract

**Fig. 1:**
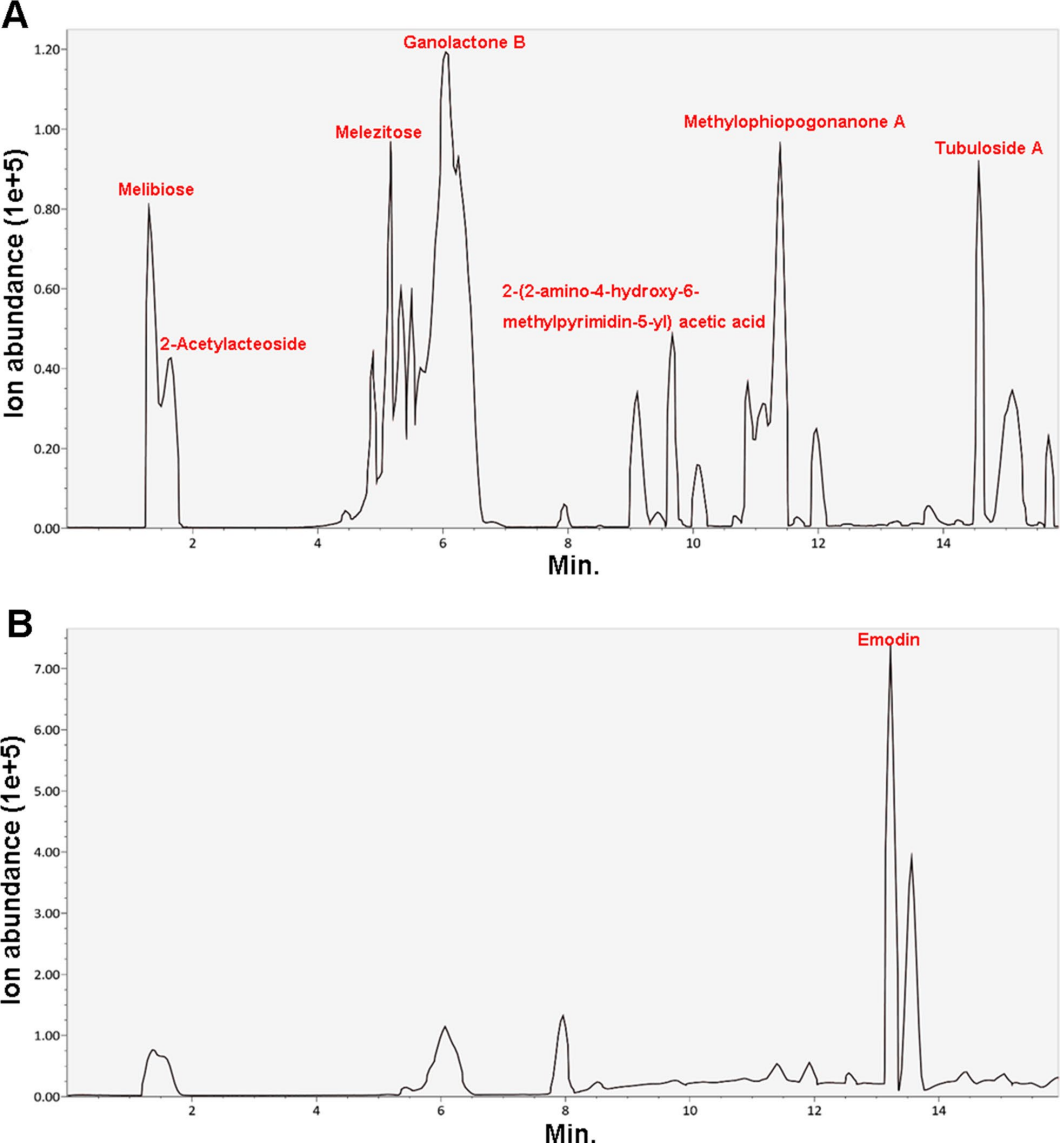
Chemical composition of *Juglans regia* L. extract. The total ion chromatograms of *Juglans regia* L extract by UPLC-MS technique. **a** Chromatogram in positive ionization mode; **b** chromatogram in negative ionization mode. *UPLC-MS* ultra-performance liquid chromatography-mass spectrometry

We confirmed the chemical composition of JRL using ultra-performance liquid chromatography–mass spectrometry (UPLC-MS). In the chromatographic profile of JRL, melibiose, melezitose, ganolactone B, methylophiopogonanone A, tubuloside A, and 2-Acetylacteoside were detected in positive ionization scan mode (Fig. 1a), whereas emodin was detected in negative mode (Fig. 1b).

### *Juglans regia* L. extract concentration-dependently promoted osteoblastic differentiation of hBMSCs

**Fig. 2:**
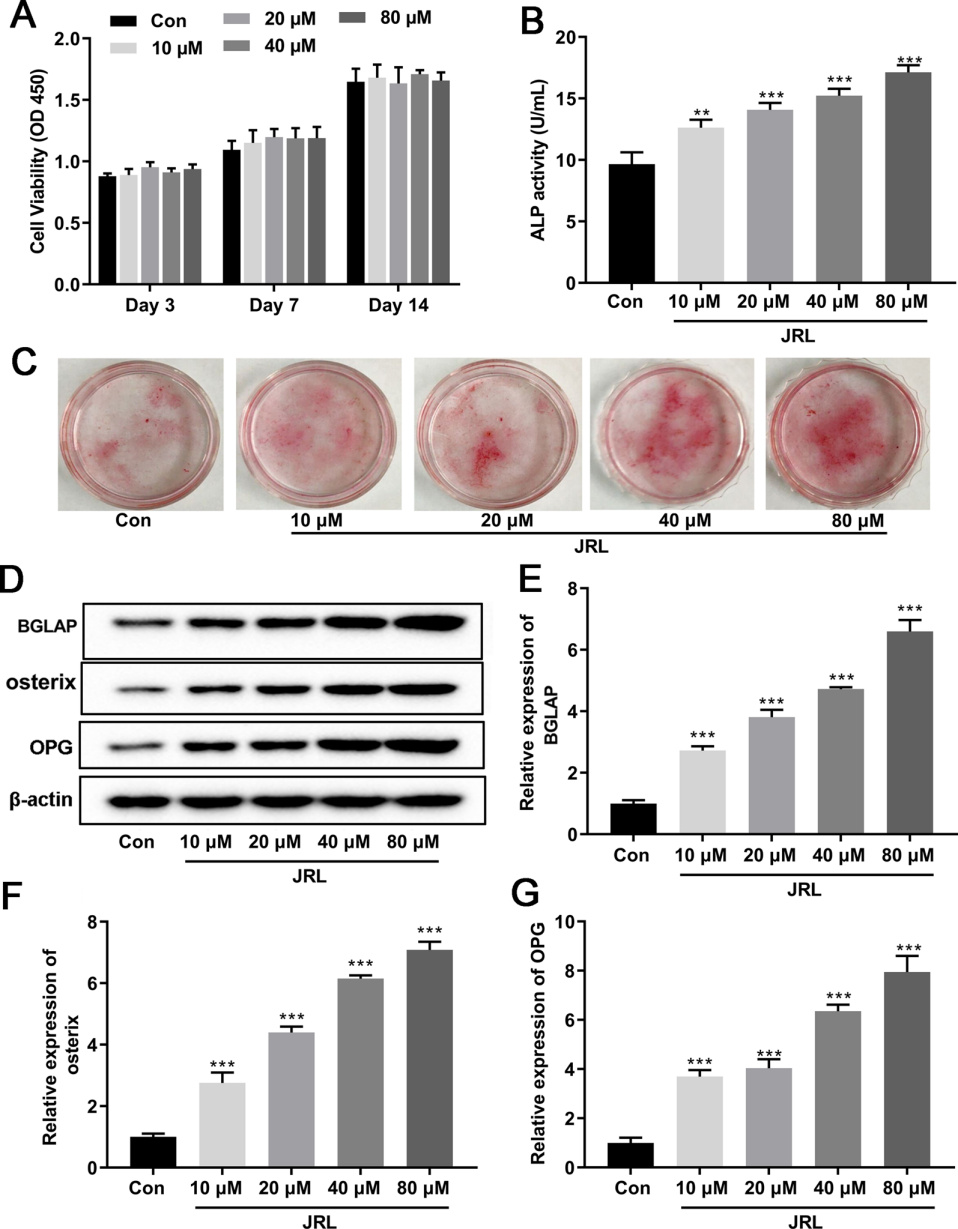
*Juglans regia* L. extract concentration-dependently promoted osteoblastic differentiation of hBMSCs. hBMSCs were treated with OIM (osteogenic induction medium, including 10 mmol/L β-glycerophosphate, 10 nmol/L dexamethasone, and 10 μg/mL ascorbicacid) and different concentrations of *Juglans regia* L extract (10, 20, 40, or 80 μM) for 1–14 days. **a** The cell viability was determined using CCK-8 solution for the cells treated by different concentration of JRL extract (10, 20, 40, or 80 μM). **b** Alkaline phosphatase (ALP) activity of hBMSCs was detected by a commercial ALP assay kit. **c** hBMSCs were treated with different concentrations of JRL, fixed and stained with Alizarin red. **d**–**g** The protein levels of Osteocalcin (BGLAP), Osterix, and osteoprotegerin (OPG) were tested by Western blot analysis. β-actin is a loading control. Data were represented as mean ± SD from at least three independent experiments performed for each assay. **P < 0.01 vs control group, ***P < 0.001 vs control group

Firstly, hBMSCs were incubated in OIM with or without different concentrations of JRL (10, 20, 40, or 80 μM) extract for 1–14 days to ascertain the effect of JRL extract on cell proliferation. According to the results of the CCK-8 assay, different concentrations of JRL extract had no significant effect on cell proliferation at different time points (Fig. 2a). To clarify the effect of JRL extract on osteoblastic differentiation, hBMSCs were cultured in OIM medium or OIM medium supplemented with JRL extract (10, 20, 40, or 80 μM) for 7 days. The quantitative analysis of the ALP activity assay evidenced that JRL dose-dependently enhanced osteogenic activity with the maximal effect observed for the 80 μM concentration (Fig. 2b). This result was also consistent with the outcome of Alizarin red staining (Fig. 2c). In addition, as shown in Fig. 2d–h, the expression of osteogenic differentiation marker genes BGLAP, osterix, and osteoprotegerin (OPG) was significantly increased induced by JRL extract (10, 20, 40, or 80 μM) treatment. All these data implied that JRL extract concentration-dependently accelerated osteoblastic differentiation of hBMSCs.

### *Juglans regia* L. extract enhanced cell autophagy of hBMSCs

**Fig. 3:**
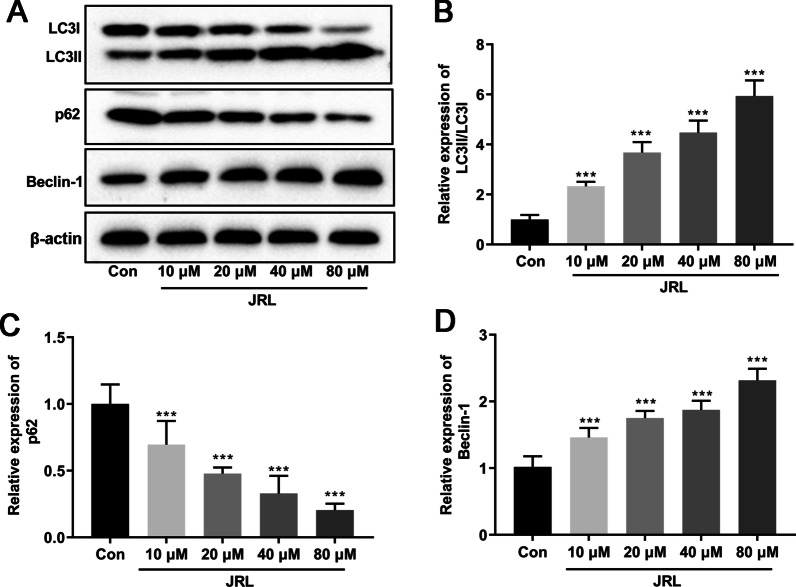
*Juglans regia* L. extract enhanced cell autophagy of hBMSCs. hBMSCs were treated with OIM (osteogenic induction medium, including 10 mmol/L β-glycerophosphate, 10 nmol/L dexamethasone, and 10 μg/mL ascorbic acid) and different concentrations of *Juglans regia* L. extract (10, 20, 40, or 80 μM) for 7 days. The protein levels of LC3II (**a** and **b**), LC3I (**a** and **b**), Beclin-1 (**a** and **d**), and p62 (**a** and **c**) were tested by Western blot analysis. β-actin is a loading control. Data were represented as mean ± SD from at least three independent experiments performed for each assay. ***P < 0.001 vs control group

Interestingly, it has been reported that autophagy plays a key role in osteoblast differentiation [24]. We thus further explored the effect of *Juglans regia* L. extract on autophagy in differentiated hBMSCs by Western blot. As shown in Fig. 3a–d, *Juglans regia* L. extract obviously enhanced autophagy of hBMSCs, as evidenced by the increases of LC3II/LC3I ratio and Beclin1 expression, as well as the attenuated p62 expression. These data demonstrated that the autophagy of hBMSCs was promoted by *Juglans regia* L. extract.

### *Juglans regia* L. extract promoted the activation BMP2/Smad/Runx2 and Wnt/β-catenin signaling pathways in hBMSCs

**Fig. 4:**
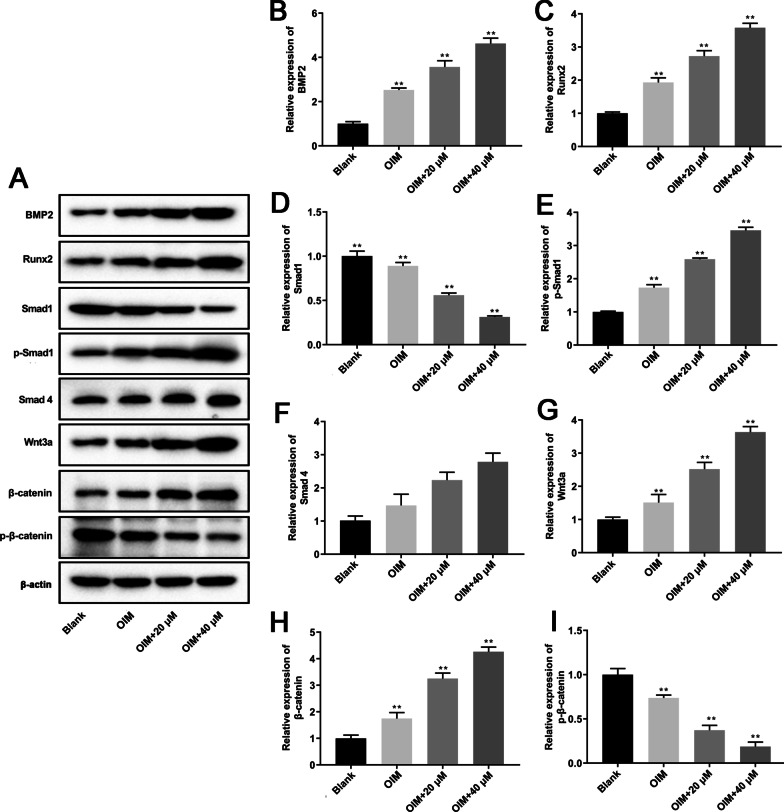
*Juglans regia* L. extract promoted the activation BMP2 and Wnt/β-catenin signaling pathways in hBMSCs. hBMSCs were treated with OIM (osteogenic induction medium, including 10 mmol/L β-glycerophosphate, 10 nmol/L dexamethasone, and 10 μg/mL ascorbic acid) and JRL extract (20 μM and 40 μM) for 7 days. The protein levels of BMP2 (**a** and **b**), Runx2 (**a** and **c**), Smad1 (**a** and **d**), p-Smad1 (**a** and **e**), Smad4 (**a** and **f**), Wnt3a (**a** and **g**), β-catenin (**a** and **h**), and p-β-catenin (**a** and **i**) were tested by Western blot analysis. β-actin is a loading control. Data were represented as mean ± SD from at least three independent experiments performed for each assay. *P < 0.05 vs control group, **P < 0.01 vs control group

Previous studies reported that BMP2/Smad/Runx2 and Wnt/β-catenin signaling pathways cooperatively regulate cytoskeletal dynamics and osteogenesis [25]. Therefore, we tested the activation effect of JRL extract treatment on the BMP2/Smad/Runx2 and Wnt/β-catenin signal pathways in hBMSCs. As shown in Fig. 4a–i, 20 μM and 40 μM JRL extract treatment significantly promoted BMP2, Runx2, p-Smad1, Wnt3a, and β-catenin expression in hBMSCs cultured with OIM medium. As expected, the expression of Smad1 and p-β-catenin was dramatically decreased by JRL extract treatment compared with OIM-cultured group.

**Fig. 5:**
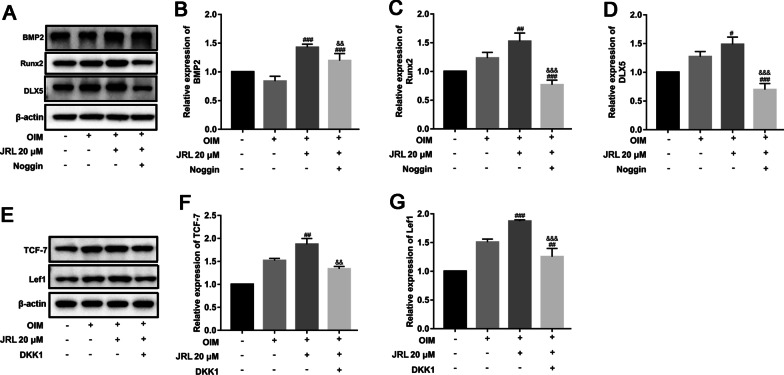
*Juglans regia* L. extract promoted the expression downstream genes of BMP2 and Wnt/β-catenin signaling pathways in hBMSCs. hBMSCs were treated with 2 mg/mL Dickkopf-1 (DKK1) or 100 ng/mL Noggin for 24 h and then treated with OIM (osteogenic induction medium, including 10 mmol/L β-glycerophosphate, 10 nmol/L dexamethasone, and 10 μg/mL ascorbic acid) and JRL extract (20 μM) for 7 days. The protein levels of BMP2 (**a** and **b**), Runx2 (**a** and **c**), DLX5 (**a** and **d**), TCF-7 (**e** and **f**) and Lef1 (**e** and **g**) were tested by Western blot analysis. β-actin is a loading control. Data were represented as mean ± SD from at least three independent experiments performed for each assay. ^#^*P* < 0.05 vs OIM-cultured group, ^##^*P* < 0.01 vs OIM-cultured group, ^###^*P* < 0.001 vs OIM-cultured group, ^&&^*P* < 0.01 vs OIM + JRL extract-cultured group, ^&&&^*P* < 0.001 vs OIM + JRL extract-cultured group

hBMSCs were then treated with OIM, 20 μM JRL extract and Noggin/DKK1 to determine the effects of JRL extract on expression of target genes of the BMP2 and Wnt/β-catenin pathways. Distal-less5 (DLX5) and Runx2 are two downstream transcription factors of BMP2 pathway. Our data showed that the expressions of BMP2, DLX5, and Runx2 were also increased in OIM and JRL extract-cultured hBMSCs. Moreover, these increased levels were substantially decreased by Noggin (Fig. 5a–d). In addition, TCF7 and Lef1 are known to be effectors of Wnt/β-catenin pathway. Compared with OIM-cultured group, the treatment of 20 μM JRL extract significantly stimulated the expression of the TCF7 and Lef1, which was reversed by DKK1 treatment (Fig. 5e–g). In general, we concluded that JRL extract stimulated the expression of key molecules of BMP2/Smad/Runx2 and Wnt/β-catenin signaling pathways.

### *Juglans regia* L. extract increased the expression of osteogenic genes and cell autophagy through the BMP2/Smad/Runx2 and Wnt/β-catenin pathways in hBMSCs

**Fig. 6:**
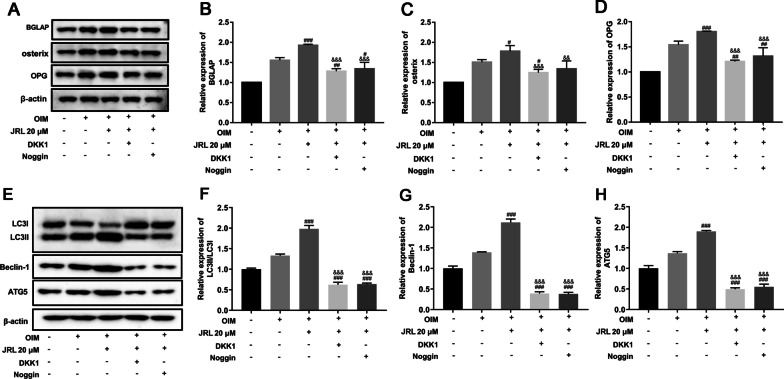
*Juglans regia* L. extract increased the expression of osteogenic genes and cell autophagy through the BMP2/Smad/Runx2 and Wnt/β-catenin pathways in hBMSCs. hBMSCs were treated with 2 mg/mL Dickkopf-1 (DKK1) or 100 ng/mL Noggin for 24 h and then treated with OIM (osteogenic induction medium, including 10 mmol/L β-glycerophosphate, 10 nmol/L dexamethasone, and 10 μg/mL ascorbic acid) and JRL extract (20 μM) for 7 days. The protein levels of BGLAP (**a** and **b**), osterin (**a** and **c**), OPG (**a** and **d**), LC3II (**e** and **f**), LC3I (**e** and **f**), Beclin-1 (**e** and **g**), and ATG5 (**e** and **h**) were tested by Western blot analysis. β-actin is a loading control. Data were represented as mean ± SD from at least three independent experiments performed for each assay. ^#^*P* < 0.05 vs OIM-cultured group, ^###^*P* < 0.001 vs OIM-cultured group, ^&&^*P* < 0.01 vs OIM + JRL extract-cultured group, ^&&&^*P* < 0.001 vs OIM + JRL extract-cultured group

Finally, we examined whether the BMP2/Smad/Runx2 and Wnt/β-catenin signaling pathways mediate the regulation of osteogenic gene expression and cell autophagy induced by JRL extract. Runx2 is an essential transcription factor for osteoblast differentiation, which was regulated by BMP2/Smad and DLX5 [26]. Meanwhile, it has been widely reported that Runx2 can trigger the expression of major bone matrix genes including SPP1, COL1, and BGLAP [27]. We found that JRL extract significantly enhanced the protein levels of the BGLAP, oxterix, and OPG compared with OIM-cultured group (Fig. 6a–d). Nevertheless, DKK1 and Noggin considerably reversed these changes (Fig. 6a–d). At last, we determined the autophagy level by Western blot and the results showed that JRL extract promoted the autophagy process, indicated by the elevated LC3-II/LC3-I ratio, Beclin1, and ATG5 expression (Fig. 6e–h). However, this augmentation was blocked by DKK1 and Noggin treatment (Fig. 6e–h).

## Discussion

JRL extract mainly contains naphthoquinone, tannin, flavone, terpene, tocopherols, steroids, and other components [28, 29]. The pharmacological activities are anti-tumor[30], antibacterial[31], cardiovascular protection[32], and so on. A high-fat diet containing whole JRL decreased the tumor size and growth rate of prostate cancer rat [33]. Previous studies showed that different concentrations JRL extract had a protective effect on cell apoptosis. Pretreatment with the male flower of JRL 80 µg/mL significantly protected against UVB-induced DNA damage and loss of mitochondrial membrane potential (MMP) in human epidermal keratinocytes [34]. JRL extract (1.5%, 3%, or 6%) induced a slow rise of intracellular calcium and finally modulated microglial activation, implying that it might be effective in neurodegeneration [35]. In the present study, we firstly investigated the effect of JRL extract (10, 20, 40, or 80 μM) pretreatment on cell viability of hBMSCs, and we confirmed different concentrations of JRL extract had no significant effect on cell proliferation, suggesting that JRL extract treated hBMSCs had reliable safety. Furthermore, the biological functions of JRL extracts, including anti-inflammatory and antioxidant, have been extensively studied. JRL was found to have the highest content of antioxidants including tocopherols among all the studied seeds and nuts [36]. JRL extract possesses significant free radical scavenging activity, and its pretreatment could inhibited expression of TNF-α, IL-1, IL-6, NF-κB, COX-2, ROS generation, and lipid peroxidation in HaCaT cells [37]. Importantly, JRL methanolic extract and ellagic acid reduced inflammation in human aorta endothelial cells and osteoblastic activity in KS483 osteoblasts [22]. Our data showed that JRL extract pretreatment promoted the expression of osteogenic genes, including osteocalcin, ALP, BGLAP, osterix, and osteoprotegerin (OPG). Interestingly, previous study demonstrated that autophagy could positively regulate osteogenesis of BMSCs [38], and we showed that JRL extract might participate in the promotion of osteogenesis via autophagy induction.

Mechanically, we found that JRL extract stimulated BMP and Wnt/β-catenin signaling pathways in hBMSCs. The BMP2/Smad signaling pathway plays an important role in the osteogenic differentiation of BMSCs [39]. BMP2 binds to receptors on the cell membrane and activates phosphorylation of Smad1, Smad5 or Smad8 in the cells [40]. The activated-Smad can be transferred from the cytoplasm into the nucleus to activate the transcription of specific target genes, including Runx2 [41]. Transcription factors Runx2 is a member of Runx family, which plays a crucial role in the growth and development of bones. It was reported that Runx2 was highly expressed in osteoblast cell lines and regulated the expression of bone specific matrix proteins (OCN, ALP, osterix, OPG, SPP1, COL1 and osteocalcin), thereby promoting the differentiation of osteoblasts [42]. In the process of bone formation, the activation of BMP2/Smad/Runx2 signaling pathway is regulated by various natural product, including total flavonoids of rhizoma drynariae [43], flavonoids [44], salidroside [45], and cibotium barometz [46]. In addition, BMP2 is a mediator of Wnt/β-catenin signaling in osteoblasts, while BMP2 expression could be induced by Wnt/β-catenin signaling [25]. Studies showed that deactivation of Wnt/β-catenin signaling inhibited bone formation and osteoblast differentiation via up-regulation of transcription factors such as TCF-7, Lef1, and c-JUN [47, 48]. β-catenin deletion blocked osteoclast precursor proliferation and prevented osteoclast differentiation, both causing osteopetrosis [49]. Other study confirmed that in mouse models with the deletion of the Wnt receptor gene, Frizzled 8 (Fzd8), bone tissues and cells displayed loss of bone mass, normal bone formation, and increased of osteoclast formationin [50]. And nitric oxide synthesis could regulate osteoblastic differentiation of adipo-derived stem cells by modulation of Wnt/β-catenin signaling [51]. In vivo study proved that osthole, a coumarin-like derivative extracted from Chinese herbs, activated osteoblast differentiation and bone formation through β-catenin/BMP2 signaling[52]. Meanwhile, kirenol enhanced osteoblast differentiation by activating of BMP2 and Wnt/β-catenin signaling pathways in MC3T3-E1 cells [53]. In addition, emerging literature suggested Wnt/β-catenin signaling pathway was also associated with cell autophagy in distraction osteogenesis [54]. Similarly, in our study, we found that JRL extract stimulated osteoblast differentiation and cell autophagy of hBMSCs through crosstalk between BMP2/Smad/Runx2 and Wnt/β-catenin signaling pathways.

## Conclusions

In this study, we firstly explored the effects and mechanisms of JRL extracts on osteogenesis of BMSCs. We concluded that JRL extract had a positive effect for the expression of osteogenic genes in hBMSCs. Meanwhile, BMP2/Smad/Runx2 and Wnt/β-catenin signaling pathways mediated osteogenesis differentiation and cell autophagy stimulated by JRL extract. Our study describes a promising therapeutic agent for patients with OP.

## Data Availability

The datasets used or analyzed during the current study are available from the corresponding author on reasonable request.

## Abbreviations

OP: Osteoporosis
JRL: Juglans regia L.
hBMSCs: Human bone marrow mesenchymal stem cells
ALP: Alkaline phosphatase
BGLAP: Osteocalcin
OPG: Osteoprotegerin
DKK1: Dickkopf-1
OIM: Osteogenic induction medium
CCK-8: Cell counting kit-8
MMP: Mitochondrial membrane potential
